# Formulation and Evaluation of Clotrimazole Mucoadhesive Vaginal Globules

**DOI:** 10.3390/gels10110716

**Published:** 2024-11-06

**Authors:** Barbara Jadach, Michalina Otworowska

**Affiliations:** Division of Industrial Pharmacy, Chair and Department of Pharmaceutical Technology, Poznan University of Medical Sciences, 3 Rokietnicka Street, 60-806 Poznań, Poland; michalina.otworowska@gmail.com

**Keywords:** globules, hydrogel base, mucoadhesion, clotrimazole

## Abstract

The aim of this study was to prepare vaginal suppositories with mucoadhesive properties to prolong the action of antifungal component clotrimazole (CLO). This was achieved by preparing vaginal pessaries on a hydrophilic gel base composed of gelatin and gelatin enriched with PEG 400 (in a 1:1 ratio), and then checking the properties of the obtained vaginal drugs. The prepared globules, containing 100 mg of CLO, were characterized in terms of mass and swelling index, organoleptic analysis was also prepared. In addition, a texture analysis and a study of the dissolution of clotrimazole were performed. On the basis of the obtained results, it was concluded that the modification of the composition of the gelatin–glycerin base by the addition of macrogol had a positive effect on the mucoadhesive properties of the globules. In addition, due to the presence of PEG 400, the globules were stiffer. It was also observed that the presence of CLO reduces the value of the force needed for compression during the texture analysis study. Comparing the CLO release profiles of the prepared globules and commercially available clotrimazole tablets, the release profile for the globules was slower than for the tablets, which indicates the possibility of using mucoadhesive globules as a form of a drug that releases the medicinal substance more slowly at the site of administration.

## 1. Introduction

Vulvo-vaginal candidiasis (VVC) is a fungal infection of the female genital organs caused by the growth of fungi from the *Candida* family. It is estimated that approximately 75% of all women will experience vaginal candidiasis at least once in their lives, and approximately 5% suffer from recurrent candidiasis [1,2,3]. Therefore, the topical treatment of vaginal yeast infection may be a rational treatment choice. A very useful and widely used drug is clotrimazole (CLO). It is a drug from the azole class of synthetic antifungals, discovered in 1960 by Karl Hienz Buchel. Azoles are the largest class of drugs that have antifungal properties [4,5,6,7]. The groups of these compounds can be divided based on their chemical structure into imidazoles and triazoles. Clotrimazole belongs to the first subgroup. It is mainly used to treat *Candida albicans* and other fungal infections. Due to its usefulness, it has been placed on the World Health Organization’s list of essential medicines and is sold as a generic drug worldwide [4,5,8,9].

Although there are many antifungal agents on the pharmaceutical market with clotrimazole [2,4,6], these drugs cannot reach high concentrations at the site of infection due to rapid removal from the vagina, low volumes of vaginal mucus, and high anatomical variability. Therefore, the use of polymers with mucoadhesive properties that can interact with the mucous membrane is currently a very popular topic among researchers [1,10,11]. By using such polymers, longer contact of the drug with the site of action can be achieved, ensuring controlled release, as well as repeatability and predictability [1,2,8,10].

The use of mucoadhesion provides many benefits; thanks to this phenomenon, the ability to control drug release by controlling the location of drug delivery to the point of contact with the mucosa has been established. Another advantage is that mucous membranes are characterized by rapid absorption and high blood flow, which results in a faster circulation rate through the blood vessels. Another important aspect is the fact that the use of this type of preparation is more comfortable for the patient. To date, many mucoadhesive systems have been studied and drugs have been developed for various therapeutic applications, such as diabetes, gum diseases, dry mouth, and aphthous ulcers [1,11,12,13]. Preparations using the phenomenon of mucoadhesion can be formulated as tablets, lozenges, rings, gels, viscous solutions, suspensions, and sprays (depending on where you want to obtain the effect and how you want to administer the drug). Polymers are materials that exhibit mucoadhesive properties [10,12,14,15]. The main division of polymers is based on the type of bond they form. We can distinguish between polymers that form non-covalent bonds and those that form covalent bonds. One example is gelatin—a denatured fibrous protein obtained from collagen connective tissue as a result of partial hydrolysis [16]. Chemically, it consists of 18 varieties of complex amino acids. The main sources of gelatin available on the market are pork skin and cartilage (46%), bovine skin and bones (29.4%), and other sources (1.5%) [17,18]. It is a natural biopolymer obtained as a result of collagen hydrolysis. The properties of the obtained product depend on the technological process used for the pretreatment of the animal material before collagen extraction [10]. Under alkaline conditions, amide groups of asparagine and glutamine are transformed into free carboxylic groups, while in the acidic process, these groups remain unchanged. It is also important that gelatin produced with the use of acidic hydrolysis is similar to untreated collagen [16,17]. The chemical properties of gelatin can be characterized through proximate composition, amino acid composition, molecular weight distribution, and pH [16]. When the gelatin is in solution form, it is free to act either as an acid or base. Gelatin possesses an amphoteric property in which the molecules are both positively and negatively charged with an isoelectric point in the range of pH 9–5, depending on the raw material and production process [16]. It can provide bioadhesion due to having a massive amount of hydrogen bond-forming moieties, such as carboxyl (–COOH), amine (–NH_2_) and hydroxyl (–OH), in its molecular structure [17].

The physical and mechanical properties of gelatin depend mainly on molecule interactions, such as hydrogen bonding and Van der Waals forces. The gel strength of commercial gelatin generally ranges from 100 g to 300 g, with a gel strength of 250–260 g as the most preferable. Besides gel strength, viscosity is considered one of the crucial physical attributes of gelatin. Low-viscosity gelatin produces short and brittle gels, while high-viscosity gelatin possesses tougher and extensible gels [16,17]. Gelatin exhibits poor mechanical properties and rapid rates of dissolution and degradation in physiological environments [17].

This polymer is biocompatible, biodegradable, and non-immunogenic. It is used in the medical field as a matrix for implants, scaffolds, device coatings, wound dressings, tissue adhesives, hemostats, and sealants, and also in many different forms of drugs as a base material (e.g., the production of hard and soft capsule shells), as a gelling agent, film-forming or coating agent, viscosity enhancer, and suspending agent, as well as a binder in the formulation of pastes, pastilles, suppositories, and tablets [10,16,17].

Vaginal preparations have evolved from a local form of therapy to a broader route of drug delivery for the treatment and prevention of disease. All innovative therapeutic options are based on the unique anatomical and physiological features of the female reproductive organ. Intravaginal drug delivery systems are used primarily in the treatment of vaginal and vulvar infections and for contraception [10,15,19].

We looked through the published literature and we did not find any publications concerned directly with the use of a gelatin base for globules with clotrimazole to which we could compare our investigation. However, there are some studies concerned with vaginal tablets with CLO. Szymańska and co-workers [8] evaluated mucoadhesive vaginal tablets by using chitosan as a matrix in order to improve drug residence time compared to commercially available dosage forms with CLO. The authors concluded that chitosan can be successfully used as a mucoadhesive excipient for vaginal solid dosage forms with CLO. Kast and co-workers [11] prepared vaginal tablets with modified chitosan as the mucoadhesive agent. Their investigations showed that, with the introduction of thiol groups to the structure of chitosan, the bioadhesive properties of the polymer could be significantly improved. The addition of clotrimazole led, thereby, to a further increase in bioadhesion to the mucosal tissue. Rao and co-workers [1] tried to enhance the solubility and dissolution rate of clotrimazole via the solid dispersion technique and prepared a mucoadhesive vaginal tablet using a suitable mucoadhesive agent. They achieved good diffusivity of the drug through all the used polymer matrices, with the highest flux recorded with Hydroxypropylmethyl celulose K100M. The mucoadhesive strength was good for all polymers, with the highest adhesion observed in case of Eudragit L100.

In the present study, globules were prepared with a gelatin–glycerol base, and also, PEG400 was added to the hydrophilic base to promote the solubility of the active substance and to check the influence of this ingredient on the mucoadhesive properties and the speed of dissolution of CLO from the globules. Also, the results from the dissolution study and those with the commercially used tablet were compared.

The aim of the described research was to prepare globules with clotrimazole, which could ensure that the drug stays longer at the site of administration. The gelatin–glycerin base consists of gelatin, glycerin, and water. It is one of the oldest forms of hydrophilic base. According to the Polish Pharmacopoeia, the ratio of ingredients is as follows: 15.0% gelatin, 15.0% water, and 70.0% glycerin. This base dissolves in the secretions of body cavities at body temperature and mixes well with active substances soluble in water and glycerin. The resulting suppositories using this base are soft and flexible, so they can be used for vaginal administration. The disadvantage of the gelatin–glycerin (GG) base is that it is a hydrophilic base (with water) in which microorganisms can grow easily. To prevent this, preservatives are used. An additional disadvantage is that it is hygroscopic, so it must be protected against moisture. This base slowly dissolves in the vagina, thanks to which a prolonged release effect can be obtained [20,21].

## 2. Results and Discussion

During the research, it was possible to prepare globules using gelatin–glycerol (GG) mass, and positive effects were achieved by replacing part of the glycerol in the composition with PEG 400 (GGP). It was assumed that PEG 400 would be introduced into the suppository mass in an amount of 1:1 or 1:2 in relation to the amount of gelatin, and the amount of water and glycerol would be modified. However, in the case of a mass containing PEG 400 in a ratio of 1:2 to the amount of gelatin, such a composition could not be prepared in the form of a uniform mass; therefore, the use of such a substrate was abandoned in the further stages of the work. PEG 400 was added to the base instead of to the glycerin part, and this could be the cause. Glycerin possesses higher viscosity than PEG 400, and maybe it could influence the properties of the prepared mass. When the composition with a 1:2 ratio of gelatin to PEG 400 was used, the prepared mass did not stick in the correct forms, and it was not possible to remove the prepared globules in the proper shape after 24 h. This research included globules made from a basic gelatin–glycerol mass with the addition of PEG 400 in a 1:1 ratio to the amount of gelatin used.

### 2.1. Morphological Analysis of Prepared Vaginal Suppositories

**Figure 1:**
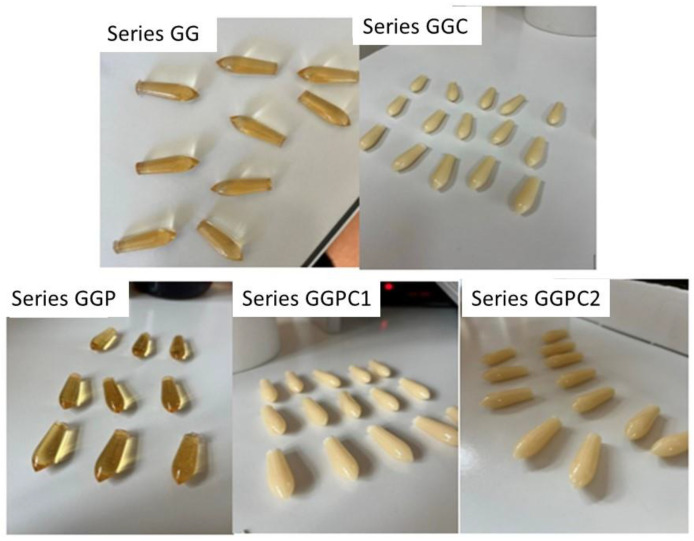
Appearance of prepared series of globules.

The analysis included control globules (GG, GGP), globules with suspended CLO (GGC, GGPC1), and globules containing partially dissolved CLO (GGPC2). It was noticed that all globules were similar in size due to being poured into the same mold (Figure 1). All prepared forms of the drug were very pliable and soft. They did not show significant differences in the visual assessment.

Differences were only noticed in appearance. The globules prepared without the active substance were more transparent, which is due to the fact that CLO is a white powder that gives the globules a different color. In the case of GGC and GGCP1, no major differences in appearance were noticed. In the case of the GGPC2 series, a darker color was observed than in the case of the GGC and GGPC1 series.

### 2.2. Mass Uniformity Study

The results in Table 1 present individual masses, means, standard deviations (SD) and coefficients of variation (RSD) for the uniformity test. It should be emphasized that the observed differences in the water content were statistically insignificant.

All globules were prepared in forms of the same volume; however, because of the differences in the density of the used ingredients, some differences in the masses of the prepared globules were expected. The displacement value, which is usually calculated during the preparation of suppositories, was not calculated. The uniformity mass study showed almost the same masses for the control (placebo) suppositories and the suppositories with clotrimazole; so, in our opinion, this calculation was not needed and provided no further information. It showed that, in the case of the globules without the active substance, the series prepared with the addition of PEG 400 had a slightly higher mass. This may be due to the fact that macrogol has a higher density than glycerin and constituted the largest part of the GG globules. The higher-mass globules with the active substance were those built on the gelatin–glycerin base with the addition of PEG 400, which was added together with glycerin, and CLO was added at the end. This may be due to the fact that the CLO was suspended and not dissolved, as in the case of GGPC2.

### 2.3. Swelling Study

**Figure 2:**
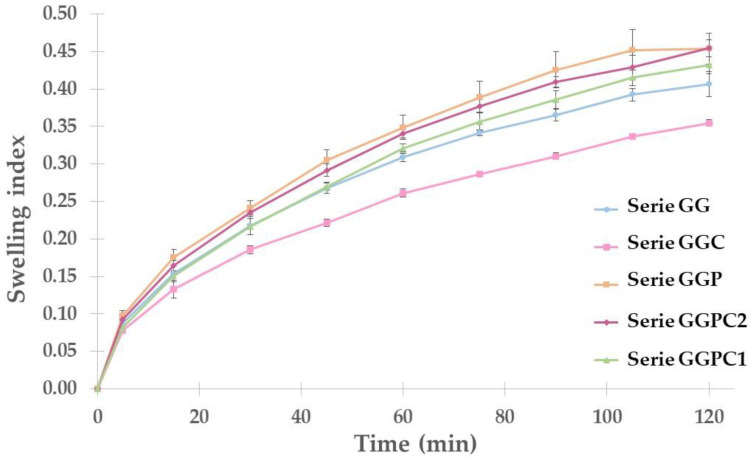
Swelling profiles of prepared globules (mean value ±SD from *n* = 3).

The results of the swelling test are presented in Figure 2 with swelling profiles. The swelling behavior of a polymer is essential for its cohesiveness with the water uptake of the dosage form [11]. For all series, no weight loss was observed throughout the study, i.e., there was no damage to the globules. It can be observed that the longer the test time, the higher the swelling index. The most significant difference in swelling behavior was observed within the first 30 min of the test.

When comparing the series of control suppositories, it can be seen that the series with the addition of PEG 400 has a higher swelling index; however, the observed differences are not statistically significant. This may suggest that by improving the traditional gelatin–glycerin globules, the mucoadhesiveness of this form of the drug was increased. Also, analyzing the results for the globules with the active substance, it can be seen that modifying the globules with the addition of macrogol has a positive effect on the swelling index. The highest swelling index was obtained for the series containing CLO, which was mixed with PEG 400, and finally added to the prepared gelatin–glycerin mass. It can be concluded that the addition of macrogol increases the mucoadhesiveness of gelatin–glycerin globules. The swelling index has also been examined in other publications. In the case of the research conducted by Szymańska et al. [8], the maximum swelling index is approximately 5, and after approximately 70 min, the tablets fail, as suggested by the descending curves. The globules obtained in present study are characterized by significantly lower swelling rates, which may suggest that they do not exhibit such a strong mucoadhesion phenomenon as chitosan tablets [8]. Analyzing the results of the swelling index for the lactose-based tablets, it can be concluded that the swelling index values are higher than in the case of the prepared globules. Unfortunately, the duration of the study differed significantly: in the case of the tablets, the study lasted as much as 8 h; therefore, comparing the results is subjective [1].

### 2.4. Texture Analysis

**Figure 3:**
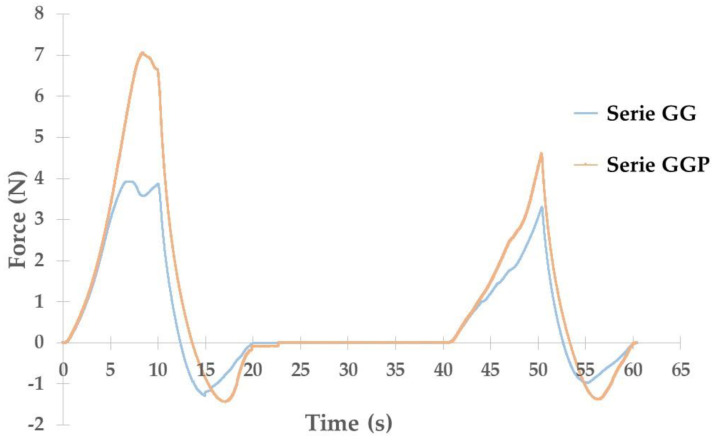
Texture profile of control suppositories (mean value from *n* = 3).

In this study, using a texture analyzer, a comparison of the mechanical properties of the prepared globules was performed in order to observe the differences between two series of control globules (Figure 3). Such a comparison allowed us to assess whether changing the gelatin solvent would affect the mechanical properties of the globules. It was observed that the matrices containing the active substance turned out to be less durable than those without the drug, which also may indicate an interaction of the active substance with the matrix components.

**Figure 4:**
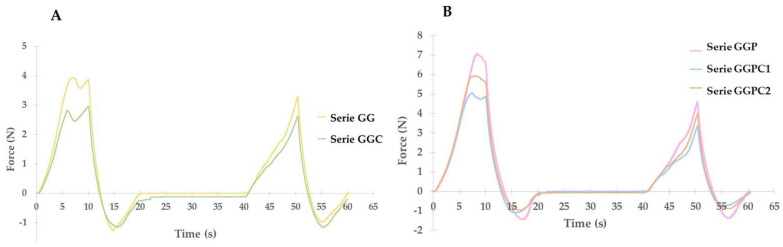
Influence of CLO on the texture profile of globules (mean value from *n* = 3). (**A**) Globules on GG base; (**B**) globules on GGP base.

The figure shows that replacing part of the glycerin portion with PEG 400 resulted in an increase in the stiffness of the globules. An increase in the force needed for the first compression from approximately 4 to 7 N was observed. In the next stage of the test (second compression), the required force did not differ that much: for globules made of traditional GG mass it was about 3.5 N, while for globules with the addition of PEG 400 (GGP base) it reached a value of about 4.5 N. It can also be observed that, for the GG base, the differences in both compression stages do not differ significantly: they reach approximately 4 and 3.5 N, while for the base with the addition of PEG 400, these two stages require forces of 7 and 4.5 N, respectively. It can be seen that the presence of PEG 400 stiffens the structure of the suppository base. At the same time, it can be said that the GG mass is a base that maintains its mechanical properties regardless of external mechanical influences. Further comparisons assessing the impact of the presence of CLO suspended in the medium on its mechanical properties are shown in Figure 4.

Analyzing the results of the globules on the GG mass, it can be seen that the addition of the active substance reduces the force needed for the first compression from approximately 4 to 3 N. During the second compression, the value of the required force does not differ much; for control globules, it is approximately 3.5 N, and for CLO globules it reaches 2.5 N. For both series, the first compression stage is not very different from the second compression. It can be concluded that the addition of the active substance slightly reduces the stiffness of the suppository base. In the case of suppositories with the addition of PEG 400, a decrease in the force needed for the first compression for globules with the addition of the active substance was observed. The lowest force value was recorded for GGPC1 globules; this value was approximately 5 N, while for the GGPC1 series, it was approximately 6 N. In the case of globules with a GG base, for the second compression, the force that was needed was similar for all three compared series; for control globules, it was 4.5 N, for globules with CLO added at the end it was 4 N, and for globules with CLO mixed with PEG 400 it reached 3.5 N. When comparing the values of force needed for the first and second compression, significant differences can be noticed. The general trend that can be observed in the study is that the presence of active substance in each batch results in less force needed to deform the globule.

### 2.5. Release Profiles

**Figure 5:**
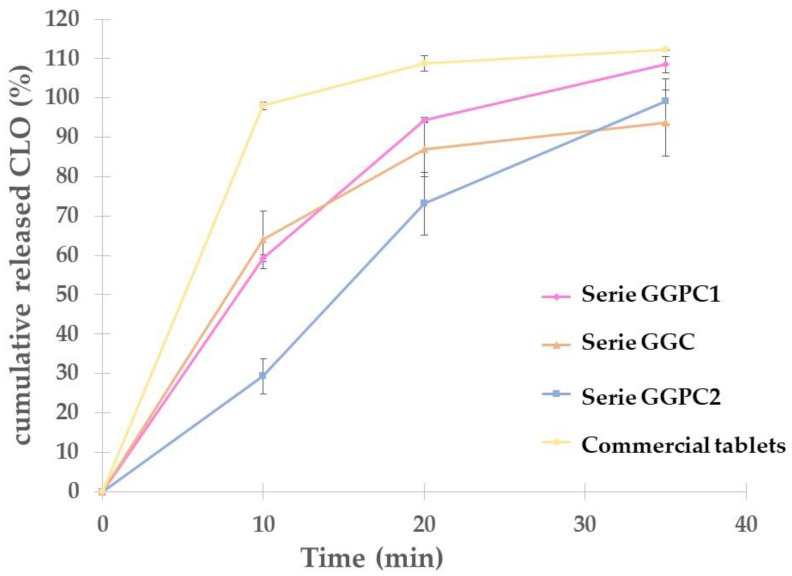
Comparison of dissolution profiles of prepared suppositories and commercial tablets (mean value ± SD from *n* = 3).

The results of the dissolution study are presented in Figure 5. The release test was only undertaken for 35 min due to the complete disintegration of the tablets and globules after this time. Comparing the release profiles of the prepared globules, it can be seen that CLO was released the slowest in the case of the GGPC2 series, and after 35 min there was almost a 100% release of the active substance. In the case of the GGC and GGPC1 series, the initial release profile was very similar.

Differences were observed after 20 min, while 85 and 95% of CLO were released from GGC and GGPC1, respectively. However, after 10 min of the investigation, only 30% of CLO was released from the GGPC2 series, while in the same time the GGPC1 series released 60% of CLO. Further, after 20 min, 72 and 95% of CLO were released from GGPC2 and GGPC1, respectively. Such a difference suggests the impact of the procedure of joining the ingredients during the preparation process. Therefore, it can be concluded that the addition of PEG 400 and mixing it with CLO before the addition of the active substance to the suppository base has a beneficial effect on the release profile. For commercial tablets with clotrimazole, the release profile differs significantly from the release profiles of the prepared globules in this study (Figure 5). The active substance was released much faster in the case of the commercially available product. After just 10 min, all of the CLO was released. The release profiles from all series of vaginal suppositories were less rapid than in the case of the tablets. The active substance was released gradually, not completely, as in the case of the tablets. The results discussed show the impact of changing the form and composition of the drug on the dissolution of CLO from vaginal preparations. By changing the formulation, a controlled and gradual release can be achieved. Additionally, it was possible to achieve the desired effect of slower release of the active substance from the prepared globules, which makes the use of this form of medicine more comfortable to use than tablets.

Kast et al., in their research [11], prepared a conjugate with clotrimazole. The conjugate was prepared from a combination of chitosan and carbodiimide. The dissolution study showed that a maximum of 12% of CLO was released after 6 h. Therefore, the developed formulation was not desirable due to the very low percentage of the active substance released. Comparing the release tests performed by Rao and Paul [1] with the results obtained in the present study, it can be concluded that in the case of lactose-based tablets, the release time was much longer (8 h), which is favorable from the point of view of the mucoadhesion phenomenon. However, when comparing the % of released CLO, higher results were obtained for the proposed globules, because for each series a result close to 100% was obtained, and in the case of the tablets, a maximum of approximately 80% was achieved [1]. Khalil [22] checked how different suppository bases affect the release of the drug substance. They used three types of substrates: a gelatin–glycerin substrate, a substrate based on Witepsol H37 and H35, and a substrate based on a mixture of PEG 400 and PEG 4000. In the case of suppositories with a traditional gelatin–glycerin base, a maximum of 80% of the active substance was released within 30 min. The time at which the maximum amount of CLO was released was similar to the results obtained in this work. Comparing the amount of substance released in the case of the globules obtained as part of our research, the amount released in our study was higher, because approximately 93% of CLO was released, and in the case of the research conducted by Khalil, this value was 80%. In the case of the macrogol mixture, the complete release time was approximately 50 min. During this time, 100% substance release was achieved. Therefore, it can be concluded that the use of PEG 400 in suppositories positively affects the release of the active substance. This is confirmed by both Khalil’s research and ours, where 100% release of the active substance was obtained in the case of globules with the addition of macrogol. For suppositories based on Witepsol, the amount of substance released did not exceed 20%, which may suggest that it is a bad base for CLO [22]. Chitosan is a mucoadhesive polymer that is nowadays very often subjected to research in order to develop new, mucoadhesive forms of drugs. Szymańska and co-workers [8] prepared chitosan-based tablets with clotrimazole and subjected them to a release study. Depending on the chitosan content, the % of clotrimazole released and the release time of the substance vary. For the formulation with the lowest polymer content, the highest results for the released substance were obtained, but the time required for complete release was 60 min. For the remaining two formulations with a higher chitosan content, longer release times were obtained, which is more favorable from the point of view of the mucoadhesion phenomenon. However, the amount of CLO released was approximately 80%. Analyzing these results and those obtained in our study, it can be concluded that in dosage forms with mucoadhesive properties and slow release of the drug substance, it is difficult to obtain 100% release.

## 3. Conclusions

Analyzing the results obtained during the evaluation of the prepared vaginal suppositories, it was concluded that the modification of standard gelatin–glycerin bases with the addition of PEG 400 did not influence the visual properties of the globules. The introduction of PEG 400 to the base influenced the mucoadhesive properties of the globules; it also made the base stiffer. On the other hand, the active substance made the base with macrogol soften. The release profiles of clotrimazole from the prepared globules were less rapid than from the commercial tablets, and for the GGPC2 series, the release was slowest, which was what was expected at the beginning of the research.

The antifungal activities test is usually required to ensure the efficacy of a formulation and make comparisons. However, the present study used a very well-known active substance without any modifications. It is also important that the preparation of mucoadhesive globules does not influence the antifungal activity of the substance. In our opinion, this part of the investigation could be missed, as the prepared globules contained widely used doses of clotrimazole.

It is also worth mentioning other perspectives; publications clearly report that the mucoadhesive effect is very desirable for vaginal forms of drugs. Thanks to this effect, it is possible to keep the drug at the “site of administration and action” for a longer time. The research proposed in this paper indicates that it is quite easy to prepare globules with such properties. However, it seems necessary to further modify the composition of the base, which will significantly slow down the release of the active substance at the site of application. This could make the drug’s action more effective and efficient.

## 4. Materials and Methods

### 4.1. Materials

Clotriamzole (Lot No 56NTH), glycerin (Lot No 094004), potassium hydrogen phosphate (Lot No 01115/01/20), and sodium lauryl sulfate (Lot No 409VNR) were purchased from Pol Aura (Poznan, Poland). Acetonitryl (Lot No102644151) and acetate buffer pH 4.5 concentrate (Lot No 1732801861) were purchased from Avantor Performance Materials (Gliwice, Poland). Methanol (Lot No 1265/08/21) was purchased from VWR Chemicals (Gdańsk, Poland). PEG 400 (Lot No BCBL6294V) was purchased from Chempur (Piekary Śląskie, Poland). Gelatin (Lot No 060) was purchased from Cykoria S.A (Wierzchowice, Poland).

### 4.2. Preparation of Globules

To prepare the base of the suppositories, first, gelatin was weighed, combined with the appropriate amount of water, and left for 15 min to swell. The next step was to add glycerol or a mixture of glycerol and PEG 400 and place the prepared mixture in a water bath (temp. 80 °C). After liquefying the gelatin, the water loss was replenished and the prepared mass was poured into a mold. The first globules were a basic gelatin–glycerol substrate, while PEG 400 was introduced into subsequent series of globules in an amount of 1:1 or 1:2 in relation to the amount of gelatin, and the amount of water and glycerol was modified. Due to the difficulty of obtaining a mass containing PEG 400 in a 1:2 ratio to the amount of gelatin, it was decided not to use such a substrate in further stages of the work. In the next stage, three batches with the active substance were prepared (Table 2).

The first product produced was gelatin–glycerin globules (GGC). After preparing the base, clotrimazole was added and mixed gently to avoid aeration of the mass, and the globules were poured into molds. The next two series of suppositories contained the addition of PEG 400, but they differed in the method of preparation. In the case of the GGPC1 series, macrogol was added during the preparation of the base and CLO was added to the prepared base. However, in the preparation of the GGPC2 series, clotrimazole was first triturated with PEG 400, and in this form was added to the previously prepared gelatin–glycerin mass. All prepared series of globules were stored in a cool place after hardening. A number of tests were carried out to enable their characterization and comparison.

### 4.3. Appearance and Mass Uniformity of the Globules

Each series of prepared globules was inspected visually for shape and uniformity of color. From each series, 10 globules were randomly selected and weighed to determine uniformity mass, and the standard deviation and coefficient of variation for each batch were calculated.

### 4.4. Water Uptake and Swelling Index

In order to determine the swelling index of the globules from each series, 3 randomly selected globules were weighed and then placed in beakers containing approximately 25 mL of acetate buffer with a pH of 6. The liquid was prepared using a concentrate for preparing an acetate buffer with a pH of 4.8, to which sodium hydroxide was added to increase the pH of the fluid. At set time intervals (i.e., 5, 15, 30, 45, 60, 75, 90, 105, and 120 min), each globule was removed, dried, weighed, and then placed back in the beaker with the buffer. The wetting (swelling) coefficient was calculated based on Formula (1), where SI is swelling index; W_1_ is the mass of the globules at the beginning of the study (in mg); and W_2_ is the mass of the globules at a time point after staying in the buffer (in mg). The test results are presented in Figure 2.

(1) $$\mathrm{SI}=\frac{W_{2}-W_{1\;}}{W_{1}}$$

### 4.5. Texture Analysis of Globules

This analysis provides information on the behavior of globules subjected to stress. The test was performed using a Shimadzu AGS-X Series texturometer (Shimadzu, Kyoto, Japan). The investigation allowed us to compare the mechanical properties of the prepared globules. Each type of prepared suppository was tested three times. A full-scale 1000 N sensor was used. The parameters of the study are summarized below in Table 3.

In the study, the globules were subjected to force by moving the sensor from above to a depth of 10 mm. During the study, the force with which the globules affected the moving sensor was measured. The obtained results were analyzed using TrapeziumX software (version 1.5.2; Shimadzu Corporation, Kyoto, Japan) and graphed as dependence of force on time.

### 4.6. Dissolution Study

The release study of clotrimazole from the prepared globules was performed in a basket apparatus, while for the commercially available CLO tablets, a paddle apparatus was used (Agilent 708-DS, 850-DS; Santa Klara, CA, USA). The conditions used during the study were chosen based on descriptions of vaginal tablets from different publications [1,8].

The test medium used was a buffer with a pH of 6, with the addition of 0.4% SDS to maintain *sink* conditions. The test was carried out in 900 mL of medium for each series (n = 3) at a temperature of 37 °C for 35 min. The rotation speed of the baskets and paddles was set to 100 rpm. We chose 100 rpm for the movement of the paddles or rotation of the baskets based on Pharmacopeial regulations, which determine such a speed for baskets as the regular one. We started with the investigation of globules and later decided to compare them with commercial tablets, so the same speed was chosen for the paddle evaluation. The study involved placing one pessary/tablet in each of three baskets/beakers (n = 3). During the test, 5 mL samples were automatically taken from each beaker at appropriate time points: 10, 20, 35 min. After each collection, the chambers were filled with the appropriate amount of fresh buffer. The amount of released CLO was determined using a validated HPLC method with UV–Vis detection. The results are presented as cumulative release profiles.

### 4.7. HPLC Analysis

The determination of CLO amount during the tests was performed using the HPLC method with isocratic elution and UV–Vis spectrophotometric detection [23]. The study was performed using a Nexera-i LC-2040C liquid chromatograph (Shimadzu, Kyoto, Japan) by setting the following parameters: mobile phase: 55%—acetonitrile solution, 45%—phosphate-buffered solution with pH = 9; mobile-phase flow rate: 0.75 mL/min; sample (injection) volume: 20 μL; column temperature: 40 °C; wavelength detection: 210 nm. A column with the following parameters was used for the stationary phase: Kinetex pheomenex (Shim-pol); Phenyl-Hexyl 100; pores: 2.6 μm, dimensions: 100 × 3 mm. Validation was performed in accordance with ICH guidelines. During validation, parameters such as linearity, precision, accuracy, and the assessment of method specificity were determined.

### 4.8. Statistical Analysis

The data were analyzed using Statistica software ver. 13 (TIBCO Software Inc. Palo Alto, CA, USA). Analysis of variance (ANOVAs) was used to determine the statistical significance between samples. The a priori level of significance was *p* < 0.05. The experiments were completed in triplicate (while mass deviation *n* = 10) and the results are represented as mean ± standard deviation (SD).

## Figures and Tables

**Table 1:** Mass uniformity of vaginal suppositories (mean value ± SD, from *n* = 10).

Series	Mass ± SD (g)	RSD (%)
GG	2.63 ± 0.06	2.1
GGC	2.63 ± 0.05	1.9
GGP	2.70 ± 0.04	1.5
GGPC1	2.70 ± 0.05	1.7
GGPC2	2.69 ± 0.03	1.2

**Table 2:** Ingredients used for the preparation of the globule bases.

Serie	Gelatin	Water	Glycerin 85%	PEG 400	CLO *
	% in the Base				Y/N
GG	15	15	70	-	N
GGC	15	15	70	-	Y
GGP	15	25	45	15	N
GGPC1	15	25	45	15	Y
GGPC2	15	25	45	15	Y

* Presence (Y) or absence (N) of CLO in globules.

**Table 3:** Parameters for texture analysis of globules.

	First Compression	First Recall	Interval	Second Compression	Second Recall
	Moving down	Moving up	Stop	Moving down	Moving up
Speed of movement of sensor	60 mm/min	60 mm/min	-	60 mm/min	60 mm/min
Change point	force	force	20 s	force	force
	10 mm	10 mm		10 mm	10 mm

## Data Availability

The raw data supporting the conclusions of this article will be made available by the authors on request.
